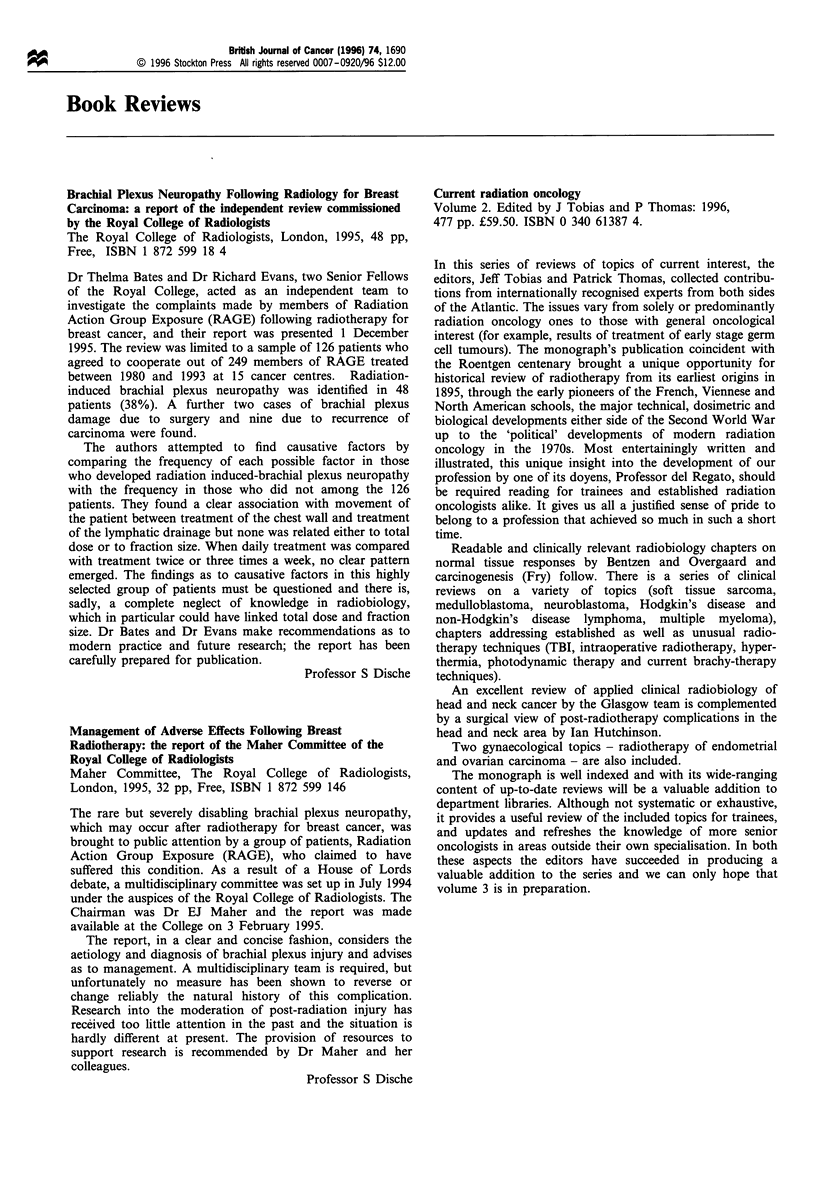# Brachial Plexus Neuropathy Following Radiology for Breast Carcinoma: a report of the independent review commissioned by the Royal College of Radiologists

**Published:** 1996-11

**Authors:** S Dische


British Journal of Cancer (1996) 74, 1690
© 1996 Stockton Press All rights reserved 0007-0920/96 $12.00

### Book Reviews

**Brachial Plexus Neuropathy Following Radiology for Breast Carcinoma: a report of the independent review commissioned by the Royal College of Radiologists**
The Royal College of Radiologists, London, 1995, 48 pp, Free, ISBN 1 872 599 18 4

Dr Thelma Bates and Dr Richard Evans, two Senior Fellows of the Royal College, acted as an independent team to investigate the complaints made by members of Radiation Action Group Exposure (RAGE) following radiotherapy for breast cancer, and their report was presented 1 December 1995. The review was limited to a sample of 126 patients who agreed to cooperate out of 249 members of RAGE treated between 1980 and 1993 at 15 cancer centres. Radiation-induced brachial plexus neuropathy was identified in 48 patients (38%). A further two cases of brachial plexus damage due to surgery and nine due to recurrence of carcinoma were found.

The authors attempted to find causative factors by comparing the frequency of each possible factor in those who developed radiation induced-brachial plexus neuropathy with the frequency in those who did not among the 126 patients. They found a clear association with movement of the patient between treatment of the chest wall and treatment of the lymphatic drainage but none was related either to total dose or to fraction size. When daily treatment was compared with treatment twice or three times a week, no clear pattern emerged. The findings as to causative factors in this highly selected group of patients must be questioned and there is, sadly, a complete neglect of knowledge in radiobiology, which in particular could have linked total dose and fraction size. Dr Bates and Dr Evans make recommendations as to modern practice and future research; the report has been carefully prepared for publication.

Professor S Dische